# Loop-mediated isothermal amplification (LAMP) colorimetric phenol red assay for rapid identification of α^0^-thalassemia: Application to population screening and prenatal diagnosis

**DOI:** 10.1371/journal.pone.0267832

**Published:** 2022-04-28

**Authors:** Wittaya Jomoui, Hataichanok Srivorakun, Siriyakorn Chansai, Supan Fucharoen

**Affiliations:** 1 Department of Pathology, Maha Chakri Sirindhorn Medical Center, Faculty of Medicine, Srinakharinwirot University, Ongkharak, Nakhon Nayok, Thailand; 2 Centre for Research and Development of Medical Diagnostic Laboratories, Faculty of Associated Medical Sciences, Khon Kaen University, Khon Kaen, Thailand; Imam Abdulrahman Bin Faisal University, SAUDI ARABIA

## Abstract

**Background:**

Identification of α^0^-thalassemia (SEA and THAI deletions) is essential in preventing and controlling of severe thalassemia diseases. We have developed the LAMP colorimetric assays for the detection of these two thalassemia defects and validated them in population screening and prenatal diagnosis.

**Methods:**

Three LAMP colorimetric assays specific for α^0^-thalassemia (SEA deletion), α^0^-thalassemia (THAI deletion) and normal DNA sequence were developed. These assays were validated on 341 subjects who had initial thalassemia screening positive and various thalassemia genotypes. Prenatal diagnosis of α^0^-thalassemia (SEA deletion) was done on 33 fetuses at risk of having Hb Bart’s hydrops fetalis syndrome.

**Results:**

The LAMP colorimetric assays for α^0^-thalassemia (SEA and THAI deletions) could be clearly interpreted by naked eyes. The assay for α^0^-thalassemia (SEA deletion) showed a 100% (62/62 x 100) sensitivity and 98.2% (274/279 x 100) specificity whereas, that of the α^0^-thalassemia (THAI deletion) showed 100% (1/1 x 100) sensitivity and 99.7% (339/340 x 100) specificity. We obtained a 100% concordant prenatal diagnosis results using LAMP assays of α^0^-thalassemia (SEA deletion) in 33 fetuses as compared to the conventional PCR analysis.

**Conclusions:**

The LAMP colorimetric assays developed are simple, rapid, and do not require sophisticated equipment. Inclusion of the LAMP tests in the existing screening protocol significantly reduce the screening cost and the molecular analysis workload, which should prove useful in the prevention and control program of hemoglobinopathies in the region.

## Introduction

α-Thalassemia is a genetic disorder caused by the absent (α^0^-thalassemia) or decreased (α^+^-thalassemia) production of α-globin chain. This α-thalassemia defect is prevalent in tropical and subtropical areas [1, 2]. In Southeast Asian countries, α-thalassemia is found at a frequency of about 30–40% [3]. A micro mapping survey in northeast Thailand has identified the prevalence of around 5.8% for α^0^-thalassemia and 25–30% for α^+^-thalassemia [4]. Two deletional forms of α^0^-thalassemia, i.e. α^0^-thalassemia (SEA deletion) or - -^SEA^ and α^0^-thalassemia (THAI deletion) or - -^THAI^, are found that are targeted for a prevention and control program in the region [4, 5]. The homozygous α^0^-thalassemia is associated with a fatal condition known as hemoglobin (Hb) Bart’s hydrops fetalis. Pregnancy with this condition has an increased risk of severe maternal complications [6, 7]. Compound heterozygosity for α^0^- and α^+^-thalassemias leads to the Hb H disease, and interaction of Hb H disease with Hb E can lead to the Hb AEBart’s and EFBart’s diseases, the thalassemia intermedia commonly encountered in the region [8, 9]. Identification of α^0^-thalassemia carrier is useful for genetic thalassemia counseling and a prevention and control program of these diseases. Initial screening could be done using the osmotic fragility (OF) test, reduced MCV & MCH, Hb H inclusion test, and the presence of Hb Bart’s or ζ-globin chain in peripheral blood detected by the immunochromatographic assay [10–15]. These screening tests are useful since they are simple, cheap, straightforward, and have high sensitivity despite having relatively high false positives due to other mild forms of thalassemia or iron deficiency anemia. Individuals screened positive at peripheral or small community hospitals are further investigated at Hb and DNA analyses to identify α^0^-thalassemia. Generally, this DNA analysis by gap-PCR or real-time PCR and related techniques are performed at reference centers or tertiary hospitals. This could be time-consuming and inconvenient, especially when conducted in pregnancy which needs rapid results for providing accurate genetic counseling and risk assessment for having severe thalassemia in the expecting fetuses.

A rapid molecular screening based on the loop-mediated isothermal amplification (LAMP), rolling circle amplification (RCA), and recombinase polymerase amplification (RPA) have been reported [16, 17]. This LAMP technique has been applied for the rapid detection of several diseases. The method uses a single tube amplification with isothermal. The result can be read by the naked eyes based on the colorimetric assay, lateral flow with a specific probe, or turbidity [18, 19]. Of these, a colorimetric assay with a phenol red (pH indicator) system is designed to provide a fast, precise visual detection of amplification based on the production of protons. The process occurs from the extensive LAMP reaction, producing a change in solution color from pink to yellow [20, 21]. In this study, we have developed the LAMP colorimetric assay for detection of α^0^-thalassemia (SEA and THAI deletion) and evaluated it in a population screening and prenatal diagnosis of Hb Bart’s hydrops fetalis syndrome.

## Materials and methods

### Subjects and specimens

Ethical approval of this study was obtained from the Institutional Review Board of Srinakharinwirot University, Thailand (SWUEC-E-338/2563). This study is not reporting a retrospective data of medical records of the patients. Some archival DNA samples of the patients were obtained from routine services for setting up the LAMP assays, all patients’ data were fully anonymized before we accessed them. According to the IRB of Srinakharinwirot University, in this case, no additional informed consent is required. Left-over DNA specimens were recruited from our ongoing thalassemia screening program at the Department of Pathology, Maha Chakri Sirindhorn Medical Center, Faculty of Medicine, Srinakharinwirot University, Thailand. 341 specimens with positive initial screening using reduced MCV & MCH and dichlorophenolindophenol (DCIP) test and Hb analysis were obtained. In addition, a total of 33 families at risk of having fetuses with Hb Bart’s hydrops fetalis syndrome were also recruited.

### Routine thalassemia testing

The hematological parameters were obtained on hematology automation (Sysmex XN3000, Sysmex, Kobe, Japan). The DCIP test for Hb E was performed using a KKU-DCIP kit. Hb analysis was done using capillary electrophoresis (Capillarys 2; Sebia, Lisses, France) [10, 22]. Identifications of the seven deletional α-thalassemia (α^0^-thalassemia: SEA, THAI, FIL, MED, 20.5 kb deletions, and α^+^-thalassemia: 3.7 and 4.2 kb deletions) and two common non deletional α^+^-thalassemia (Hb Constant Spring and Hb Paksé) are performed routinely using PCR and related methods described elsewhere [23, 24].

### Development of LAMP colorimetric assay

**Fig 1:**
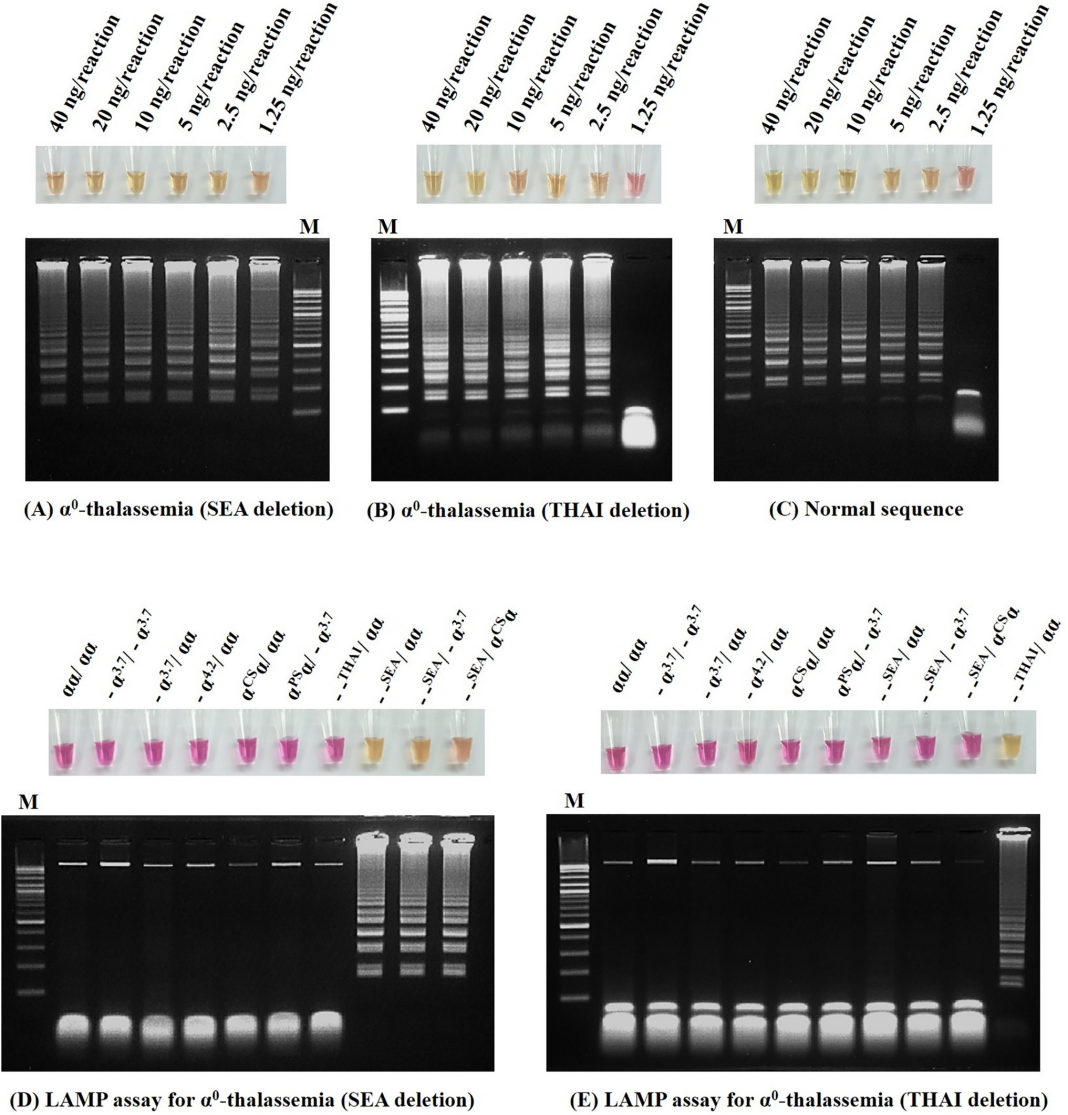
Determination of the lower limit of detection (LOD) of the developed LAMP colorimetric assays for α^0^-thalassemia (SEA and THAI deletion) and gel electrophoresis. The DNA template ranges 1.25–40 ng/reaction, including (**A**) α^0^-thalassemia (SEA deletion), (**B**) α^0^-thalassemia (THAI deletion), and (**C**) normal. Specificity of the developed LAMP colorimetric assays was demonstrated on subjects with various thalassemia genotypes as indicated for (**D**) α^0^-thalassemia (SEA deletion) and (**E**) α^0^-thalassemia (THAI deletion).

The LAMP assay for detection of α^0^-thalassemia (SEA and THAI deletions) and normal α-globin gene was developed using a colorimetric phenol red assay. All LAMP primers set in this study were designed using the software Primer Explorer V5 (http://primerexplorer.jp/lampv5e/index.html). Each set contains four primers, including F3, B3, FIP, and BIP (Table 1). As for the conventional gap-PCR, LAMP primers were designed based on the DNA deletions. Primers F3 & FIP are located on the 5’ breakpoint whereas B3 & BIP are located on the 3’ breakpoint of the corresponding deletion. However, for the normal DNA sequence primer set, we submitted the DNA sequences located nearby the α2-globin gene to the program for primers design. This study used WarmStart^®^ Colorimetric LAMP 2x Master Mix (DNA & RNA) (New England Biolabs; MA, USA). The assay contains phenol red as a pH indicator; changes the color from pink to yellow which naked eyes can observe. The LAMP mixture, temperature, and reaction duration related to α^0^-thalassemia SEA deletion, THAI deletion, and normal α-globin gene detection are summarized in Table 1. The lower limit of detection (LOD) in the developed LAMP assay was determined using a two-fold dilution (DNA template range 1.25–40 ng/reaction) as shown in (Fig 1A–1C). The specificity of the protocol was demonstrated in LAMP colorimetric and gel electrophoresis as shown in (Fig 1D and 1E).

**Table 1:** Protocols of the LAMP colorimetric assays for α^0^-thalassemia (SEA and THAI deletions), and normal DNA sequence. Sequences of the oligonucleotide primers used in each protocol and the LAMP reaction mixtures are listed.

Protocol	Primers (5’> 3’)	Chromosome 16 (NC_000016.10)	LAMP colorimetric assays
α^0^-thalassemia (SEA deletion) NC_000016.10: Del_165398–184701	**(F3)** CGATCTGGGCTCTGTGTTC **(B3)** TGGAGTGCAGTGTTGTAGTC **(FIP)** GGACGACCGAGTTCCTGCGA-GAGGGAAGGAGGGGAGAAG **(BIP)** CGCCTTGGGGAGGTTCACTT-AGCCTTGAACTCCTGGACTT	165257–165275	**Reaction mixture**: A total of 25 μl contained 2 μl of (20–50) ng/μl genomic DNA, 12.5 μl of WarmStart^®^ Colorimetric LAMP, 4.0 μl of 5M Betaine, 0.5 μl of 10 μM each primer of F3 and B3, 0.5 μl of 40 μM each primer of FIP and BIP, and the remainder distilled water. **Isothermal temperature**: 65 °C, 60 minutes
		184762–184781	
		165324–165343	
		165285–165303	
		165381–165400	
		184730–184749	
α^0^-thalassemia (THAI deletion) NC_000016.10: Del_149863–183312	**(F3)** CAGCTCTATTGAGATAAAATTCACA **(B3)** GAGTGCAAATTCCCCTGG **(FIP)** CTGTACCAAGTGGGCTGAGC-CGGTTCACCCATTTAAAGTG **(BIP)** CTACCCAGAGGTGCAGATCCA-TGAGTGGGCATGAGTCTC	149790–149814	**Reaction mixture**: A total of 25 μl contained 2 μl of (20–50) ng/μl genomic DNA, 12.5 μl of WarmStart^®^ Colorimetric LAMP, 5.0 μl of 5M Betaine, 0.5 μl of 10 μM each primer of F3 and B3, 1.0 μl of 40 μM each primer of FIP and BIP, and the remainder distilled water. **Isothermal temperature**: 65 °C, 90 minutes
		183427–183444	
		183320–183339	
		149822–149841	
		183343–183363	
		183406–183423	
Normal DNA sequence	**(F3)** TCGGTAGAGGCGGGGT **(B3)** CTTGGTGCTGGGGTACAC **(FIP)** TTGGATGGCGTTGGCGGC-GGAGCTCAGGGAGGTGGA **(BIP)** GGTGTGGGCGCTAGTGAAGC-GCAGGACCAGGTCAGTGAC	166091–166106	**Reaction mixture**: A total of 25 μl contained 2 μl of (20–50) ng/μl genomic DNA, 12.5 μl of WarmStart^®^ Colorimetric LAMP, 0.5 μl of 10 μM each primer of F3 and B3, 1.0 μl of 40 μM each primer of FIP and BIP, and the remainder distilled water. **Isothermal temperature**: 65 °C, 55 minutes
		166278–166295	
		166152–166169	
		166112–166129	
		166203–166222	
		166247–166265	

### Screening for α^0^-thalassemia (SEA and THAI deletion) using LAMP colorimetric assay

Re-screening of α^0^-thalassemia was carried out on 341 left-over DNA specimens from a routine service with various thalassemia genotypes. This was done in blinded trial with the LAMP colorimetric assays described above and the result was compared with that of the conventional gap-PCR assay.

### Prenatal diagnosis of α^0^-thalassemia using LAMP colorimetric assay

**Fig 2:**
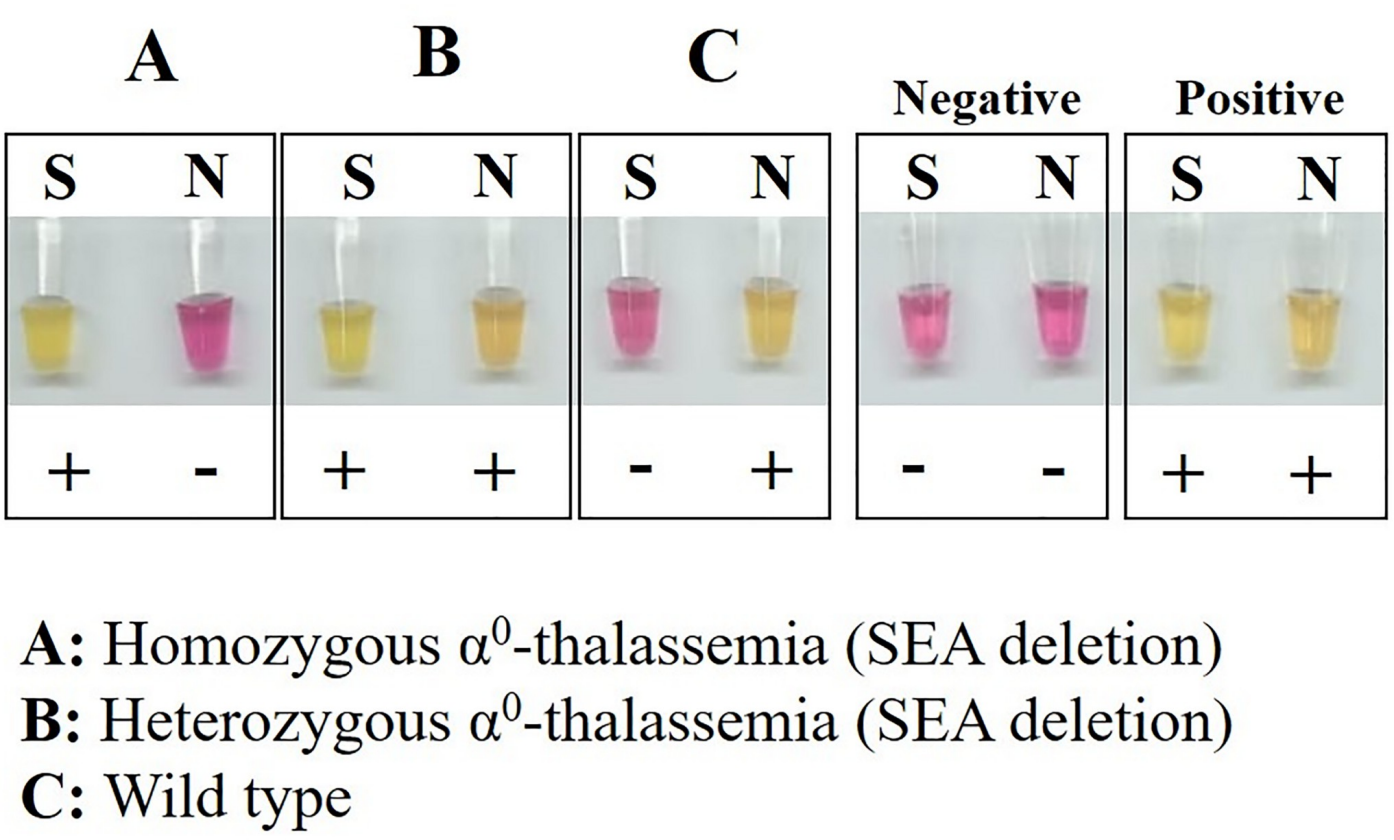
The possible genotypes of the fetus as examined using the LAMP colorimetric assays in prenatal diagnosis of Hb Bart’s hydrops fetalis syndrome (SEA deletion). A, B, and C represent homozygous α^0^-thalassemia, heterozygous α^0^-thalassemia, and normal subject, respectively. S and N indicate the LAMP colorimetric assays for α^0^-thalassemia (SEA deletion) and normal DNA sequence.

Prenatal diagnosis was done on 33 families at risk of having fetuses with Hb Bart’s hydrops fetalis syndrome caused by homozygous α^0^-thalassemia. Fetal DNA was extracted from amniotic fluid specimens [25]. Prenatal diagnosis was carried out with the developed LAMP colorimetric assay as well as the conventional gap-PCR assay. Two LAMP colorimetric assays for detection of α^0^-thalassemia (SEA deletion) and a normal DNA sequence located upstream of α-globin gene were performed (Fig 2).

## Results

Fig 1A–1C demonstrated the sensitivity of the developed LAMP colorimetric assay for detection of α^0^-thalassemia (SEA & THAI deletions) according to a LOD value. The LOD value for α^0^-thalassemia (SEA deletion) was 1.25 ng/reaction, while α^0^-thalassemia (THAI deletion) and normal α-globin gene were found to be 2.5 ng/reaction. The LAMP colorimetric assays developed showed specificity for α^0^-thalassemia (SEA & THAI deletions) and a concordant result with detection by gel electrophoresis detection in amplified LAMP product as shown in (Fig 1D and 1E).

The result of screening for α^0^-thalassemia (SEA & THAI deletions) by the LAMP colorimetric assays on the 341 subjects with various thalassemia genotypes were summarized in Table 2. This included a total of 33 genotypes and 15 phenotypic groups, as listed in the table. Among these 341 subjects, 62 carried α^0^-thalassemia (SEA deletion), including [--^SEA^/-α^3.7^, β^A^/ β^A^] (n = 3), [--^SEA^/-α^3.7^, β^E^/ β^A^] (n = 4), [--^SEA^/ αα, β^E^/ β^E^] (n = 3), [--^SEA^/ αα, β^E^/ β^A^] (n = 14), and [--^SEA^/ αα, β^A^/ β^A^] (n = 38). Only one subject carried α^0^-thalassemia (THAI deletion) [--^THAI^/ αα, β^A^/ β^A^]. The remaining 278 subjects had 26 different genotypes of α-thalassemia, β-thalassemia, and αβ-thalassemia as listed. Among these 341 subjects examined, 67 (19.6%) subjects tested positive in the LAMP colorimetric assay for α^0^-thalassemia (SEA deletion) detection. These included all 62 subjects with α^0^-thalassemia (SEA deletion) and additional positives from five subjects including [αα /αα, β^E^/ β^E^] (n = 2), [-α^3.7^/-α^3.7^, β^A^/ β^A^] (n = 1), [-α^3.7^/αα, β^A^/ β^A^] (n = 1), and [αα /αα, β^A^/ β^A^] (n = 1). The remaining 274 subjects had the negative results of the LAMP colorimetric assay for α^0^-thalassemia (SEA deletion). The results indicate a sensitivity of 100% (62/62 x 100), a specificity of 98.2% (274/279 x 100), a positive predictive value (PPV) of 92.5% (62/67 x 100), and a negative predictive value (NPV) of 100% (274/274 x 100) of the developed LAMP colorimetric assays for α^0^-thalassemia (SEA deletion) detection.

**Table 2:** Thalassemia genotypes of 341 subjects with positive (P) and negative (N) in the LAMP colorimetric assays for α^0^-thalassemia (SEA deletion and THAI deletion). P & N are positive and negative, respectively. P* indicates false positive result of the LAMP assays.

Thalassemia genotypes	Hb & DNA analysis		LAMP colorimetric assays		No
	Hb types	α, β—genotypes	SEA deletion	THAI deletion	
Hb H disease	A_2_ABart’sH	--^SEA^/-α^3.7^, β^A^/ β^A^	P	N	3
EA Bart’s disease	EABart’s	--^SEA^/-α^3.7^, β^E^/ β^A^	P	N	4
Heterozygous α^0^-thalassemia	A2A	--^SEA^/ αα, β^A^/ β^A^	P	N	38
	A2A	--^THAI^/ αα, β^A^/ β^A^	N	P	1
Heterozygous α^0^-thalassemia with Hb E	EA	--^SEA^/ αα, β^E^/ β^A^	P	N	14
	EE	--^SEA^/ αα, β^E^/ β^E^	P	N	3
Homozygous α^+^-thalassemia	A2A	-α^3.7^/-α^3.7^, β^A^/ β^A^	N	N	11
	CSA2A	α^CS^α / α^CS^α, β^A^/ β^A^	N	N	2
	A2A	-α^3.7^/-α^3.7^, β^A^/ β^A^	P*	N	1
Homozygous α^+^-thalassemia with Hb E	EA	α^3.7^/-α^3.7^, β^E^/ β^A^	N	N	10
	CSEA	α^CS^α / α^CS^α, β^E^/ β^A^	N	N	2
	EE	α^3.7^/-α^3.7^, β^E^/ β^E^	N	N	2
Compound heterozygous α^+^-thalassemia	A2A	-α^3.7^/ -α^4.2^, β^A^/ β^A^	N	N	1
	CSA2A	-α^3.7^/ α^CS^α, β^A^/ β^A^	N	N	5
	A2A	-α^3.7^/ α^PS^α, β^A^/ β^A^	N	N	1
Compound heterozygous α^+^-thalassemia Hb E	EA	-α^3.7^/ -α^4.2^, β^E^/ β^A^	N	N	1
	CSEA, EA	-α^3.7^/ α^CS^α, β^E^/ β^A^	N	N	7
	EA	-α^3.7^/ α^PS^α, β^E^/ β^A^	N	N	1
	EE	-α^3.7^/ α^CS^α, β^E^/ β^E^	N	N	1
	EE	-α^4.2^/ α^PS^α, β^E^/ β^E^	N	N	1
Heterozygous α^+^-thalassemia	A2A	-α^3.7^/ αα, β^A^/ β^A^	N	N	71
	A2A	-α^4.2^/ αα, β^A^/ β^A^	N	N	6
	CSA2A	α^CS^α / αα, β^A^/ β^A^	N	N	10
	A2A	-α^3.7^/ αα, β^A^/ β^A^	P*	N	1
Heterozygous α^+^-thalassemia with β-thalassemia trait or Hb E	A2A	-α^3.7^/ αα, β^E^/ β^A^	N	N	5
	CSEA	α^CS^α / αα, β^E^/ β^A^	N	N	1
	A2A	-α^3.7^/ αα, β^thal^/ β^A^	N	N	1
	EE	-α^3.7^/ αα, β^E^/ β^E^	N	N	11
	EE	α^CS^α / αα, β^E^/ β^E^	N	N	2
Compound heterozygous β-thalassemia and Hb E	EFA	αα / αα, β^+^/ β^E^	N	N	3
	EF	αα / αα, β^0^/ β^E^	N	N	2
Homozygous Hb E	EE	αα / αα, β^E^/ β^E^	N	N	33
	EE	αα / αα, β^E^/ β^E^	P*	N	2
	EE	αα / αα, β^E^/ β^E^	N	P*	1
Heterozygous β-thalassemia	A2A	αα / αα, β^thal^/ β^A^	N	N	10
Heterozygous Hb E	EA	αα / αα, β^E^/ β^A^	N	N	14
Normal	A2A	αα / αα, β^A^/ β^A^	N	N	58
	A2A	αα / αα, β^A^/ β^A^	P*	N	1
**Total**					**341**
**LAMP colorimetric assays for α**^**0**^**-thalassemia (SEA deletion)** Sensitivity = (62/62) × 100 = 100%. Specificity = (274/279) × 100 = 98.2%. Positive predictive value = (62/67) × 100 = 92.5%. Negative predictive value = (274/274) × 100 = 100%. **LAMP colorimetric assays for α**^**0**^**-thalassemia (THAI deletion)** Sensitivity = (1/1) × 100 = 100%. Specificity = (339/340) × 100 = 99.7%. Positive predictive value = (1/2) × 100 = 50%. Negative predictive value = (339/339) × 100 = 100%.					

We detected two subjects with positive LAMP colorimetric assays for α^0^-thalassemia (THAI deletion), one with [--^THAI^/ αα, β^A^/ β^A^] and another with [αα / αα, β^E^/ β^E^] genotypes. The remaining 339 subjects showed negative results in both LAMP colorimetric assay (THAI deletion) and conventional gap-PCR for α^0^-thalassemia (THAI deletion). Therefore, the sensitivity, specificity, PPV, NPV of the LAMP colorimetric assays for α^0^-thalassemia (THAI deletion) detection were 100%, 99.7%, 50.0%, and 100%, respectively. No false negative of α^0^-thalassemia (SEA and THAI deletions) was observed with the two developed LAMP colorimetric assays for α^0^-thalassemia (SEA and THAI deletion).

We have tested the LAMP colorimetric assay for α^0^-thalassemia (SEA deletion) in prenatal diagnosis of 33 fetuses at risk of having Hb Bart’s hydrops fetalis syndrome caused by homozygous α^0^-thalassemia. Fig 2 demonstrated this assay’s results for a homozygous α^0^-thalassemia, a heterozygous α^0^-thalassemia, and a normal fetus. Table 3 summarizes the overall results. The LAMP colorimetric assay identified 13 unaffected fetuses, 12 fetuses with heterozygous α^0^-thalassemia (SEA deletion), and 8 with homozygous α^0^-thalassemia (SEA deletion), which were 100% concordance with the results obtained at routine prenatal diagnosis made by a conventional gap-PCR assay.

**Table 3:** Prenatal diagnosis of 33 fetuses at risk of having Hb Bart’s hydrops fetalis syndrome caused by homozygous α^0^-thalassemia using the LAMP colorimetric assay for α^0^-thalassemia (SEA deletion) in comparison with a routine conventional gap-PCR.

Prenatal diagnostic results by conventional gap-PCR (no.)	LAMP colorimetric assay for α^0^-thalassemia (SEA deletion)			Number (Concordant result; %)
	SEA deletion	Normal sequence	Interpretation	
Unaffected (13)	Negative	Positive	Unaffected	13 / 13 (100)
Heterozygous α^0^-thalassemia (SEA deletion) (12)	Positive	Positive	Heterozygous α^0^-thalassemia	12 / 12 (100)
Homozygous α^0^-thalassemia (SEA deletion) (8)	Positive	Negative	Homozygous α^0^-thalassemia (Hb Bart’s hydrops fetalis)	8 / 8 (100)
**Total**				**33 / 33 (100)**

## Discussion

α^0^-Thalassemia is mostly caused by large DNA deletions removing duplicated α-globin genes. In Southeast Asia and China, the most common deletional form of α^0^-thalassemia is the SEA deletion with the estimated heterozygous frequency ranging from 4.5–14.0% [4, 7, 26]. A THAI deletion α^0^-thalassemia is also found with less frequency in the region. In addition to the homozygous α^0^-thalassemia, which is the life-threatening form, the clinical importance of α^0^-thalassemia lies in its interaction with other hemoglobinopathies with hematological and clinical phenotypes variable. Some examples can be seen in Table 2, in which it was found in association with various hemoglobinopathies and was identified at 18.5% of 341 study subjects. It is essential to provide an accurate diagnosis of these thalassemic forms. Routinely, it is recommended to do DNA analysis for α^0^-thalassemia with both SEA and THAI deletions.

We have demonstrated for the first time that our LAMP colorimetric assays for detecting these two determinants of α^0^-thalassemia (SEA & THAI deletions) are simple and could be used effectively at both population screening and prenatal diagnosis. We do realize that development of LAMP assays for all types of α-thalassemia including not only α^0^-thalassemia but also α^+^-thalassemia (3.7 kb and 4.2 kb deletions) as well as Hbs Constant Spring and Pakse’ would be best. However, the prime targets of thalassemia screening in the region are β- and α^0^-thalassemia carriers and Hb E since the three thalassemic diseases to be prevented are β-thalassemia major, Hb E-β-thalassemia and Hb Bart’s hydrops fetalis caused by homozygous α^0^-thalassemia. Hb H disease is not a target for prevention and control, and α^+^-thalassemia is not routinely investigated. If needed, this α^+^-thalassemia can be investigated using separate PCR assays.

The LAMP techniques developed require DNA of as low as 2.5 ng, resulting from the LOD values shown in Fig 1. We believe that the assays are suitable for several DNA extraction methods, i.e., phenol-chloroform, magnetic bead, and spin- column kits which normally yield a DNA concentration of more than 10 ng/μL [27]. These LAMP colorimetric assays require isothermal amplification at 65 °C, for 55–90 minutes. The results can be interpreted by naked eyes without a need for real-time quantitative analysis or gel electrophoresis of the amplified products. This makes the application of the assays in the field easy.

The LAMP assays for the detection of α^0^-thalassemia (SEA deletion) have previously been reported. Using different sets of primers, Chomean et al. have examined their LAMP assay on only 84 subjects, including 56 with wild-type and 28 carriers of α^0^-thalassemia (SEA deletion). The LAMP protocol was performed on isothermal at 60 °C for 60 minutes, based on colorimetric assay of phenol red indicator that changed color from pink to orange. They reported a sensitivity and specificity of 100%. However, no other α-thalassemia genotypes commonly found in the region were examined, and application in prenatal diagnosis was not tested either. In addition, it seems that the change of color from pink to orange is quite challenging to distinguish between negative and positive results [28]. In another study by Wang et al. [29], the LAMP assay has been developed using fluorescence color development under ultraviolet (UV) illumination. The assay was performed on isothermal at 56 °C, 45 minutes and tested on 400 samples including–SEA, -α3.7, and -α4.2, and five β-thalassemia mutations including 654M, 41/42M, −28M, 17M, and 27/28M. The sensitivity and specificity were reported to be 100% and 99.3%, respectively. No LAMP assay for the THAI deletion α^0^-thalassemia has been developed, and application in prenatal diagnosis was not tested either. In addition, the use of UV illumination for reading the testing result is not convenient in a routine setting. In contrast, our LAMP assays developed in this study for detecting α^0^-thalassemia (SEA & THAI deletions) could be interpreted by naked eyes, and the change of color from pink to yellow could be easily recognized.

**Fig 3:**
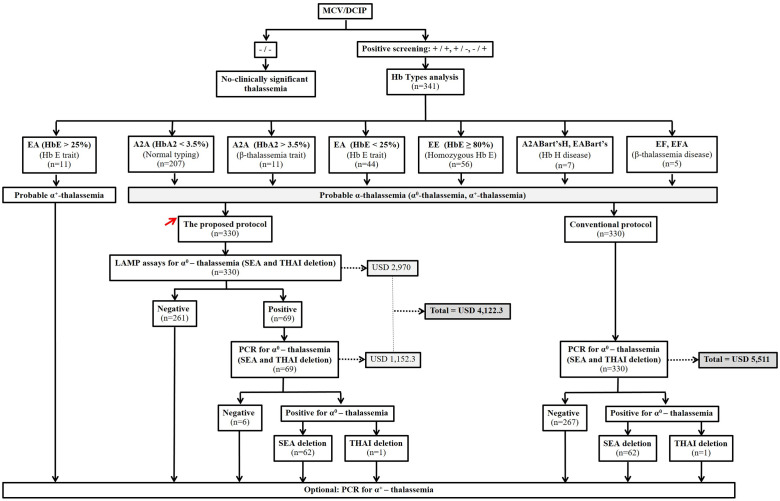
Comparison of the conventional screening protocol without LAMP colorimetric assay and the proposed screening protocol with LAMP colorimetric assay for α^0^-thalassemia. The cost-effectiveness of the two protocols was also determined on the 341 subjects. The PCR analysis of α^0^-thalassemia is needed for 330 subjects with the conventional screening protocol, whereas only 69 subjects are required for PCR analysis of α^0^-thalassemia with the proposed protocol. Break down of the 341 subjects in each screening protocol is indicated. DCIP, dichlorophenolindophenol; OF, osmotic fragility; MCV, mean corpuscular volume.

As shown in Table 2, the sensitivity of 100% of the LAMP assays for the SEA and THAI deletional α^0^-thalassemia make them suitable for rapid population screening of α^0^-thalassemia in the region. We could not define the causes of small false positives observed (5 cases for the SEA deletion and 1 case for the THAI deletion) for certain, but this seems unrelated to the thalassemia genotype. These false positives were encountered in one heterozygous α^+^-thalassemia (3.7 kb deletion), one homozygous α^+^-thalassemia (3.7 kb deletion), three homozygous Hb E, and a normal subject. This false positive may partly reflect the high sensitivity of the LAMP assay with low LOD, thus easily allowing false positive results. It has been noted that LAMP techniques are most suitable for rapid molecular screening rather than diagnostic testing [18, 19]. Based on the result in this study, we recommend using LAMP assays for rapid screening of α^0^-thalassemia before being confirmed with conventional PCR assay. However, our approach has not detected others α^+^-thalassemia (3.7 kb and 4.2 kb deletion) or non-deletional α^+^-thalassemia including Hb CS, and Hb PS. Thus, Hb H disease may be lack of information for counseling in PND. For optional, the genotypes of α^+^-thalassemia could be performed by PCR assay after the negative result from LAMP assays. As shown in Fig 3, we proposed a modified screening strategy by introducing the LAMP assays into the current screening protocol used in the region [10, 11] and compared the cost-effectiveness. The three screening steps include (OF/DCIP or MCV/DCIP), Hb analysis, and the LAMP assay. Only those with positive LAMP assay are subjected to conventional PCR analysis for α^0^- thalassemia. The strategy is rapid and straightforward and can significantly reduce the workload of conventional PCR analysis to identify α^0^- thalassemia (SEA and THAI deletion). Consequently, the cost-effectiveness of the proposed protocol based on the 341 studied subjects was determined. As shown in Fig 3, those of Hb E carriers with Hb E > 25% (n = 11) could be excluded from PCR analysis of α^0^-thalassemia. The remaining 330 subjects were further investigated. The cost per test of the developed LAMP assay and PCR analysis for α^0^-thalassemia (SEA and THAI deletion) is currently USD 9.0 and 16.7, respectively. Therefore, the cost of using a conventional protocol without LAMP assay would be **USD 5,511** (330 x 16.7). The proposed protocol with the LAMP assay would cost **USD 4,122.3** including the LAMP test (330 x 9.0 = 2,970) and the confirmatory PCR analysis of 69 LAMP positive cases (69 x 16.7 = 1,152.3). Accordingly, this could save **USD 1,388.7** for the proposed screening protocol with LAMP assay. In addition, the workload of conventional PCR analysis of α^0^-thalassemia could be reduced by 261 cases.

It is noteworthy that in addition to the rapid screening of α^0^-thalassemia, we have demonstrated for the first time that the LAMP colorimetric assay is also useful for prenatal diagnosis of Hb Bart’s hydrops fetalis caused by homozygosity of α^0^-thalassemia (SEA deletion) (Table 3). To investigate the genotype of the fetus, two LAMP colorimetric assays are needed, one for detection of α^0^-thalassemia (SEA deletion) and another for normal sequence. The prenatal diagnosis of 33 fetuses revealed a 100% concordance between the LAMP colorimetric assays and the conventional PCR analysis. No false positive and false negative was encountered. Therefore, the LAMP colorimetric assays developed could provide the best double-check system for prenatal diagnosis of α^0^-thalassemia with the conventional PCR analysis.

## Conclusion

The developed LAMP colorimetric assays are simple and rapid, do not require PCR machine and gel electrophoresis of the amplified product and the results can be interpreted by naked eyes. The assays should prove helpful in screening and prenatal diagnosis of α^0^-thalassemia, especially at a small community hospital in remote areas, before being confirmed at the reference laboratory center.

## Supporting information

S1 Raw images (PDF)
