# Supplementary material for: Loop-mediated isothermal amplification (LAMP) colorimetric phenol red assay for rapid identification of α0-thalassemia: Application to population screening and prenatal diagnosis
Source: PLoS One. 2022 Apr 28;17(4):e0267832. doi: 10.1371/journal.pone.0267832 (PMC9049341; doi:10.1371/journal.pone.0267832)
Supplement: S1 Raw images — (PDF) [file pone.0267832.s001.pdf]

Supporting Information files (S1)

Original gel pictures and images used in Figures 1 & 2

(uncropped & unadjusted)

Fig 1. Determination of the lower limit of detection (LOD) of the developed LAMP colorimetric assays for α^0-thalassemia (SEA and THAI deletion) and gel electrophoresis. The DNA template ranges 1.25-40 ng/reaction, including (**A**) α^0-thalassemia (SEA deletion), (**B**) α^0-thalassemia (THAI deletion), and (**C**) normal. Specificity of the developed LAMP colorimetric assays was demonstrated on subjects with various thalassemia genotypes as indicated for (**D**) α^0-thalassemia (SEA deletion) and (**E**) α^0-thalassemia (THAI deletion).

(A) α^0-thalassemia (SEA deletion) (lanes 1-6 from the left)

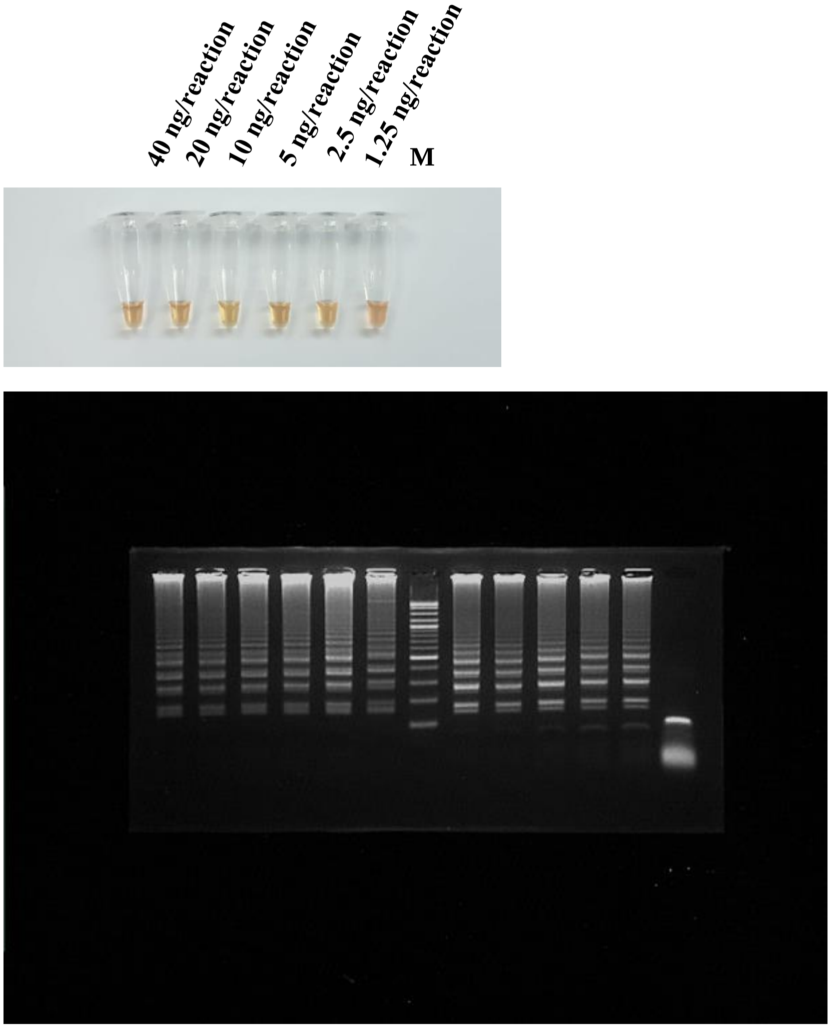

(**B**) α^0-thalassemia (THAI deletion)

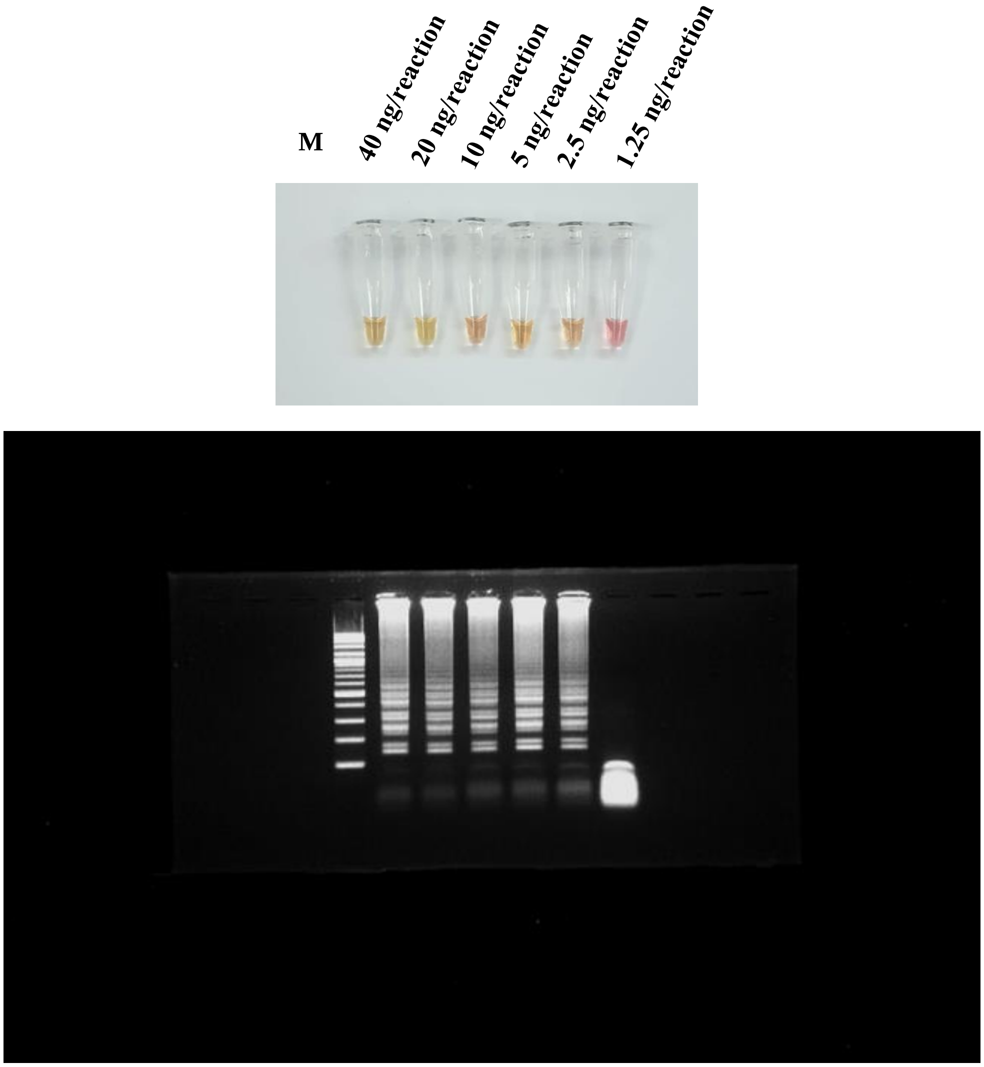

(**C**) normal (lanes 8-13)

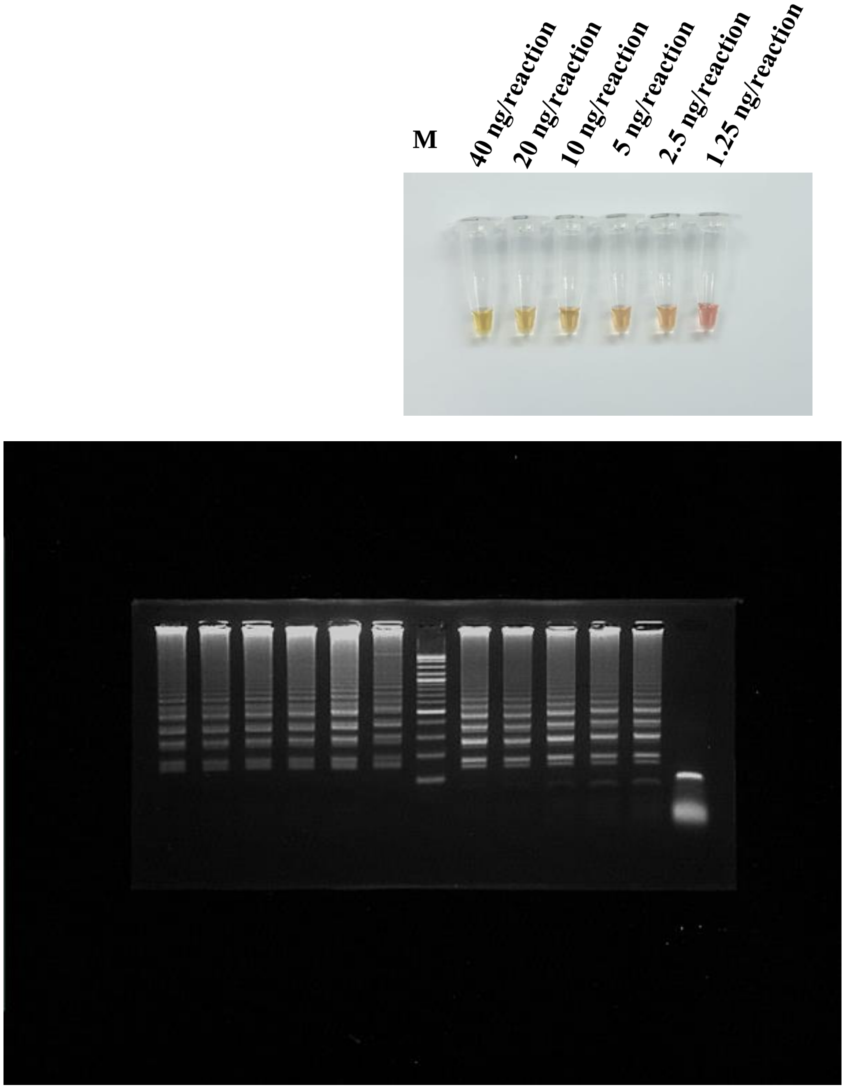

(**D**) α^0-thalassemia (SEA deletion)

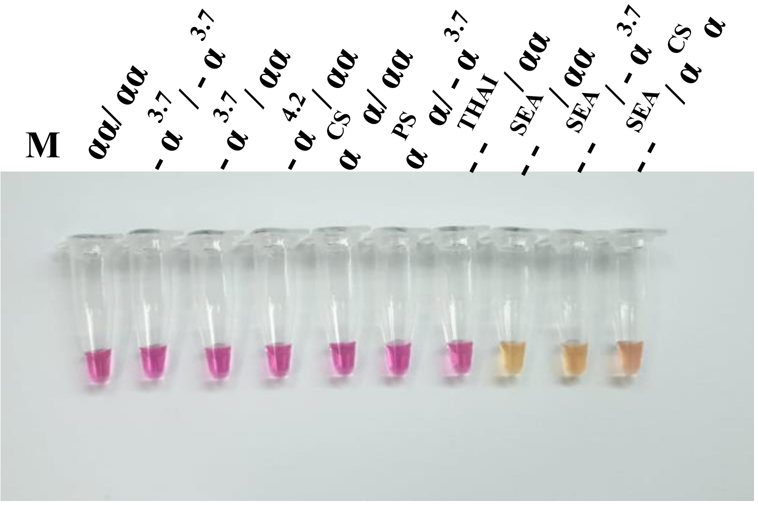

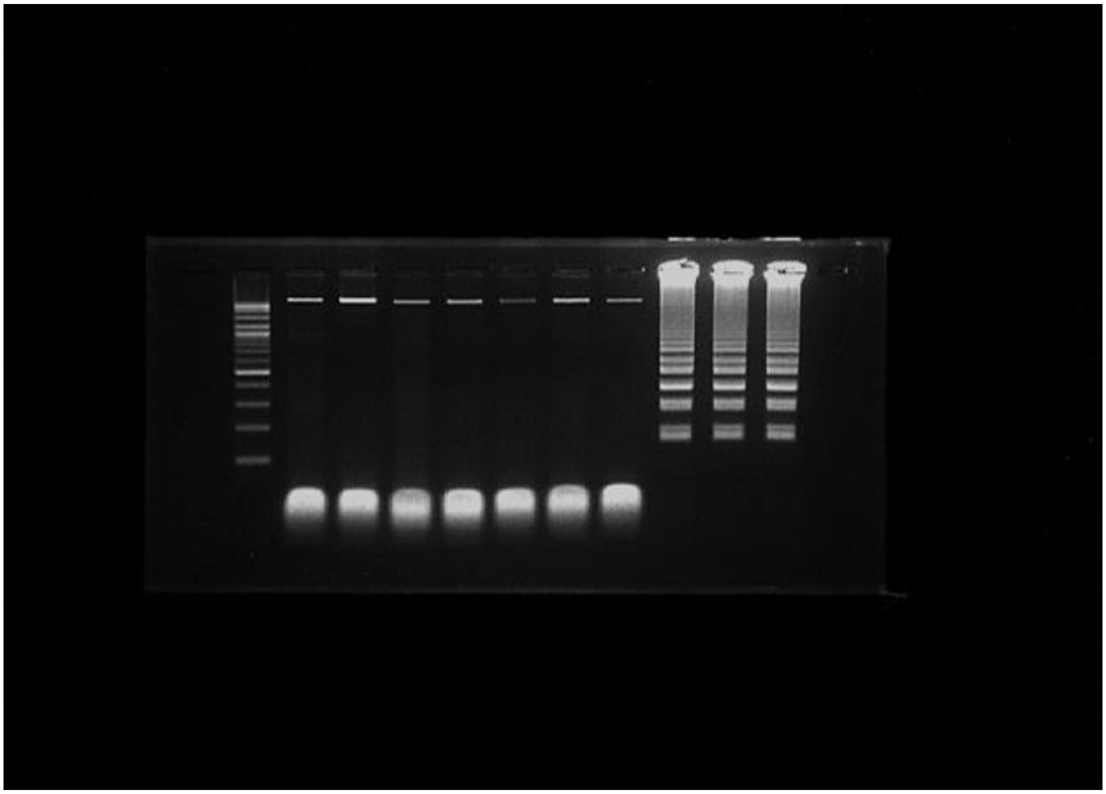

(**E**) α^0-thalassemia (THAI deletion)

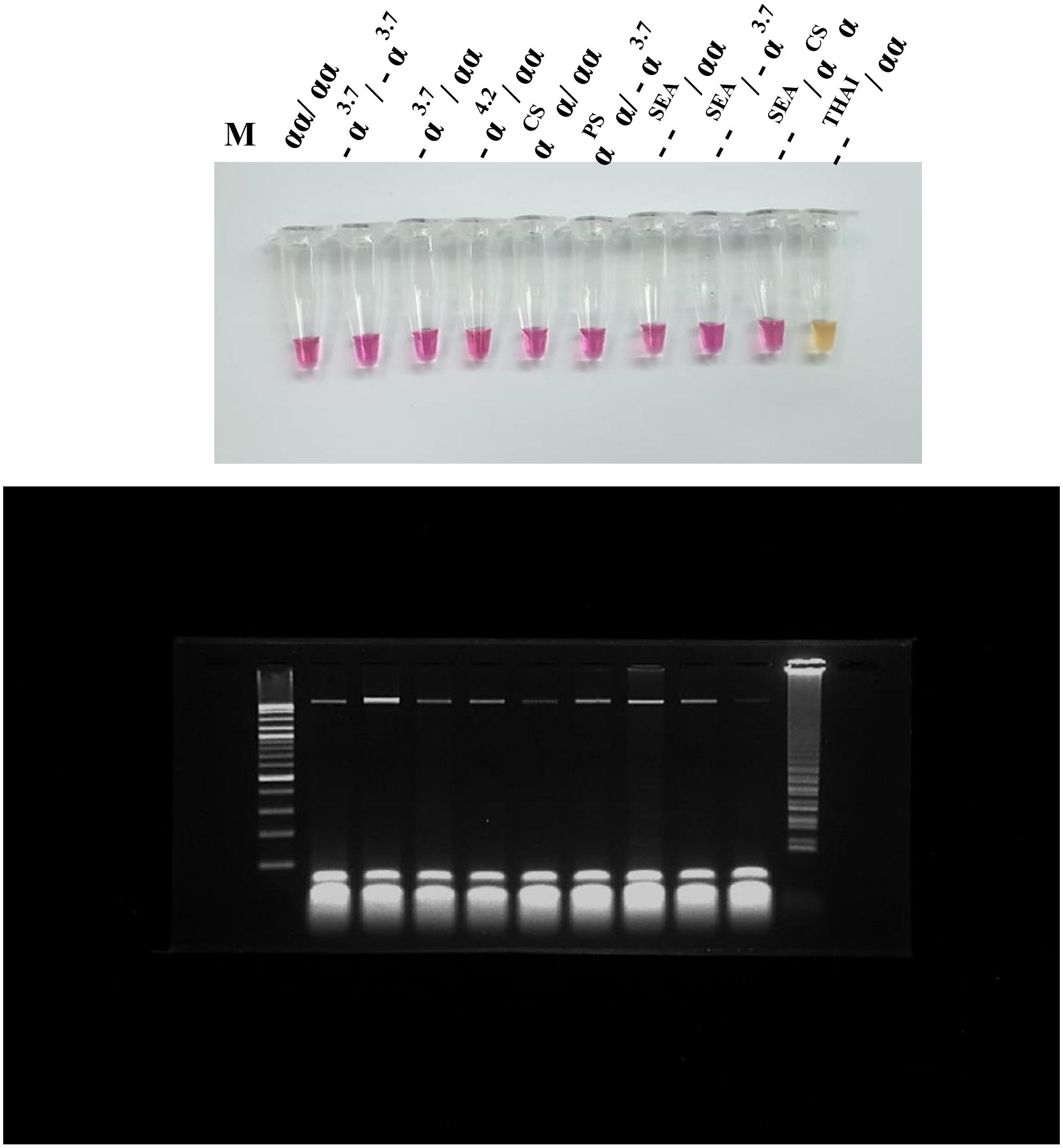

Fig 2. The possible genotypes of the fetus as examined using the LAMP colorimetric assays in prenatal diagnosis of Hb Bart's hydrops fetalis syndrome (SEA deletion). A, B, and C represent homozygous α^0-thalassemia, heterozygous α^0-thalassemia, and normal subject, respectively. S and N indicate the LAMP colorimetric assays for α^0-thalassemia (SEA deletion) and normal DNA sequence.

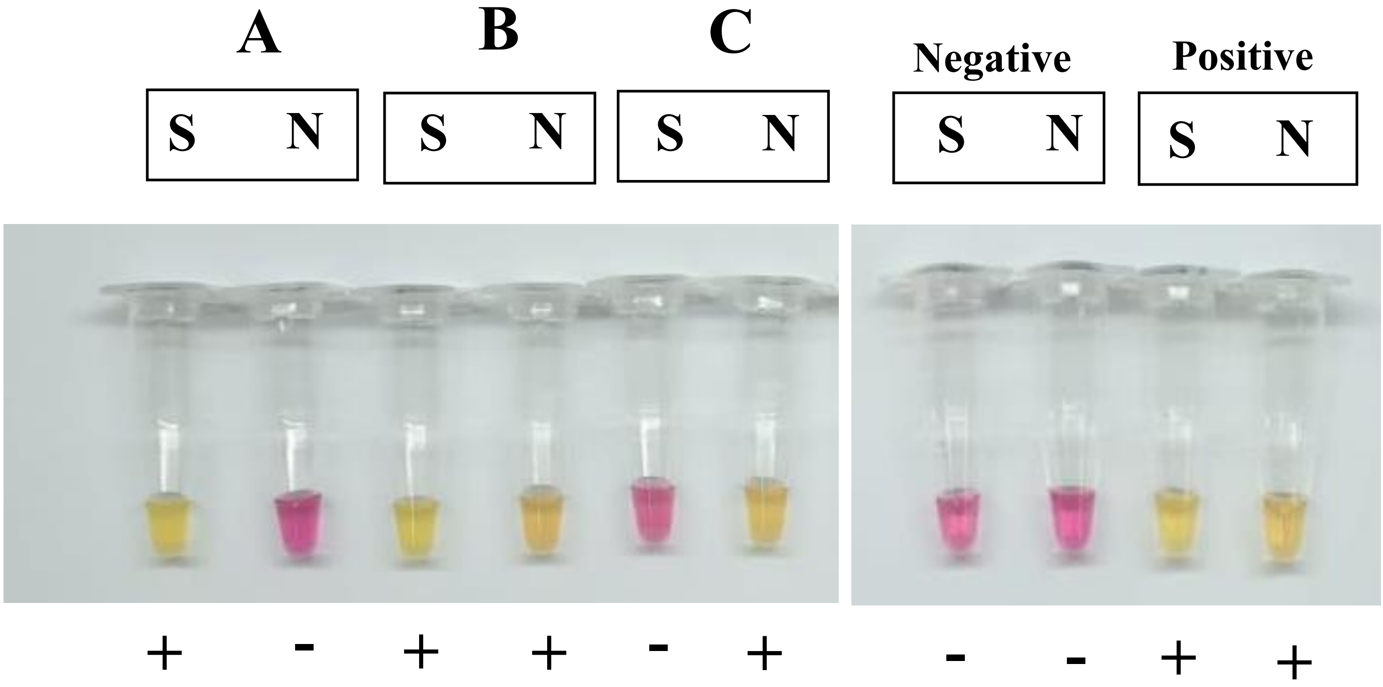

A: Homozygous α^0-thalassemia (SEA deletion)

B: Heterozygous α^0-thalassemia (SEA deletion)

C: Wild type
